# Chronic hepatitis C infection is associated with higher incidence of extrahepatic cancers in a Canadian population based cohort

**DOI:** 10.3389/fonc.2022.983238

**Published:** 2022-10-13

**Authors:** Maryam Darvishian, Terry Tang, Stanley Wong, Mawuena Binka, Amanda Yu, Maria Alvarez, Héctor Alexander Velásquez García, Prince Asumadu Adu, Dahn Jeong, Sofia Bartlett, Mohammad Karamouzian, Jean Damascene Makuza, Jason Wong, Alnoor Ramji, Ryan Woods, Mel Krajden, Naveed Janjua, Parveen Bhatti

**Affiliations:** ^1^ Cancer Prevention, BC Cancer, Vancouver, BC, Canada; ^2^ Cancer Control Research, BC Cancer Research Centre, Vancouver, BC, Canada; ^3^ Clinical Prevention Services, BC Centre for Disease Control, Vancouver, BC, Canada; ^4^ Faculty of Medicine, University of British Columbia, Vancouver, BC, Canada; ^5^ Department of Epidemiology, School of Public Health, Brown University, Providence, RI, United States; ^6^ Human Immunodeficiency Virus (HIV)/Sexually Transmitted Infection (STI) Surveillance Research Center, and World Health Organization (WHO) Collaborating Center for Human Immunodeficiency Virus (HIV) Surveillance, Institute for Futures Studies in Health, Kerman University of Medical Sciences, Kerman, Iran; ^7^ Faculty of Health Sciences, Simon Fraser University, Burnaby, BC, Canada

**Keywords:** BC-HTC, standardized incidence ratios, cancer acquisition risk, HCV, extrahepatic cancer

## Abstract

**Introduction:**

Chronic infection with hepatitis C virus (HCV) is an established risk factor for liver cancer. Although several epidemiologic studies have evaluated the risk of extrahepatic malignancies among people living with HCV, due to various study limitations, results have been heterogeneous.

**Methods:**

We used data from the British Columbia Hepatitis Testers Cohort (BC-HTC), which includes all individuals tested for HCV in the Province since 1990. We assessed hepatic and extrahepatic cancer incidence using data from BC Cancer Registry. Standardized incidence ratios (SIR) comparing to the general population of BC were calculated for each cancer site from 1990 to 2016.

**Results:**

In total, 56,823 and 1,207,357 individuals tested positive and negative for HCV, respectively. Median age at cancer diagnosis among people with and without HCV infection was 59 (interquartile range (IQR): 53-65) and 63 years (IQR: 54-74), respectively. As compared to people living without HCV, a greater proportion of people living with HCV-infection were men (66.7% vs. 44.7%, P-value <0.0001), had comorbidities (25.0% vs. 16.3%, P-value <0.0001) and were socially deprived (35.9% vs. 25.0%, P-value <0.0001). The SIRs for liver (SIR 33.09; 95% CI 29.80-36.39), anal (SIR: 2.57; 95% CI 1.52-3.63), oesophagus (SIR: 2.00; 95% CI 1.17-2.82), larynx (SIR: 3.24; 95% CI 1.21-5.27), lung (SIR: 2.20; 95% CI 1.82-2.58), and oral (SIR: 1.78; 95% CI 1.33-2.23) cancers were significantly higher among individuals living with HCV. The SIRs for bile duct and pancreatic cancers were significantly elevated among both individuals living with (SIR; 95% CI: 2.20; 1.27-3.14; 2.18; 1.57-2.79, respectively) and without HCV (SIR; 95% CI: 2.12; 1.88-2.36; 1.20; 1.11-1.28, respectively).

**Discussion/Conclusion:**

In this study, HCV infection was associated with increased incidence of several extrahepatic cancers. The elevated incidence of multiple cancers among negative HCV testers highlights the potential contributions of screening bias and increased cancer risks associated with factors driving acquisition of infection among this population compared to the general population. Early HCV diagnosis and treatment as well as public health prevention strategies are needed to reduce the risk of extrahepatic cancers among people living with HCV and potentially populations who are at higher risk of HCV infection.

## Introduction

Globally, 56.8 million individuals were living with hepatitis C virus (HCV) infection in 2020 (1). Although chronic infection with HCV is known for its causal association with liver complications like cirrhosis and hepatocellular carcinoma, its burden extends beyond the liver, including extrahepatic manifestations like non-Hodgkin’s lymphoma (NHL) (2–4). In British Columbia (BC), the number of individuals with positive HCV antibody tests has increased gradually since 2000 (5). Although highly effective direct‐acting antiviral (DAA) medications to treat HCV infection have been available in BC since 2013, 57% of individuals diagnosed with HCV in BC were among baby-boomers (born between 1945 and 1965) who carry higher risks of hepatic and extrahepatic HCV-related complications (5, 6). For instance, in a recently conducted study using the British Columbia Hepatitis Testers Cohort (BC-HTC), we found elevated risks of colorectal and pancreatic cancers among people living with HCV-infection (7). Although the causal pathways remain uncertain, oncogenic impacts of HCV proteins including oxidative stress and chronic inflammation may contribute to associations between HCV and extrahepatic cancers (8).

Although some studies have demonstrated increased incidence of other extrahepatic cancers among people living with HCV, overall, the evidence remains inconclusive. For example, Haung et al. reported elevated risk of pancreatic cancer among individuals living with HCV in a Swedish population, but this association was not observed by Abe et al. among Japanese adults (9, 10). Heterogeneous findings may be due to limitations of existing studies. Since most studies have lacked HCV negative comparison groups, risk factors associated with HCV infection, such as alcohol use and low socioeconomic status (SES), which have been also linked to elevated risks of cancer (7), are not accounted for when assessing the contribution of HCV to cancer incidence, thereby biasing study results. Second, many studies have used first HCV positive test date as the start of cohort follow-up; however, it is known that due to the asymptomatic nature of chronic HCV infection, acquisition typically occurs years before diagnosis (11). The underestimation of infection duration would likely bias risk estimates (11). To address these limitations, we used the British Columbia Hepatitis Testers Cohort (BC-HTC), to conduct a population-based cohort study assessing the incidence of various extrahepatic cancers among individuals living with HCV infection as well as a control group of individuals that tested negative for HCV.

## Methods

### Study population

The BC-HTC includes all individuals tested for HCV or HIV at the British Columbia Centre for Disease Control Public Health Laboratory (BCCDC-PHL), or individuals who have been reported to public health as confirmed cases of HCV, HBV, HIV, or active tuberculosis, since 1990 (**Supplemental Table 1**) (12). The BCCDC-PHL performs more than 95% of HCV and HIV serology testing and all confirmatory testing in British Columbia (BC). These data are linked with provincial administrative databases and registries including medical visits, hospitalizations, prescription drugs, cancer diagnoses, and death. Detailed descriptions of the BC-HTC cohort have been provided previously (7, 12, 13).

The current study included individuals who were tested for HCV up to December 31, 2015. We excluded individuals who were less than 18 years of age at cancer diagnosis, individuals who were diagnosed with cancer, hepatitis B, and/or HIV before the HCV infection diagnosis date, and individuals with missing information on age. We further excluded individuals who had a positive HCV test less than one year prior to their cancer diagnosis.

### Infection confirmation and cancer diagnosis

HCV infection status was based on HCV antibody, RNA or genotype test results, or reporting of an individual as an HCV case to public health authorities (13). Individuals with HCV infection who were later diagnosed with HBV and/or HIV infections were categorised as: HCV/HBV co-infected, HCV/HIV co-infected, and HCV/HBV/HIV triple infected. The negative test group was followed from their first negative test date up until any infection diagnosis (i.e., HCV, HBV, HIV), cancer, death, or end of the study (December 31, 2016).

The outcome of interest was cancer diagnosis, identified by International Classification of Diseases for Oncology, version 3 (ICDO-3) codes (14). Cancer diagnoses for BC-HTC participants were ascertained by linking with the BC Cancer Registry (BCCR) (7). This registry has been gold certified by the North American Association of Central Cancer Registries with a 97.1% case-capture rate (7, 15). The following cancer site groupings were used in this study: anal, bile duct, bladder, body of uterus, breast, cervix, colorectal, oesophagus, gallbladder, kidney, larynx, liver, lung, melanoma, oral, ovary, pancreas, prostate, stomach, testis, thyroid, and all other cancers (**Supplemental Table 2**) (16).

### Defining baseline dates

For individuals living with HCV, start of follow-up should be date of infection. However, in general, the exact date of HCV infection remains unknown, probably occurring years before the diagnosis date. In a previously conducted study using BC-HTC data, among 64,634 individuals who tested positive for HCV, 7064 (10.9%) individuals had multiple test dates that captured seroconversion (i.e., HCV test results changed from negative to positive) (17). The median age at time of seroconversion was 33 years. Hence, as a proxy for date of infection, we used the date of each individual’s 33^rd^ birthday. For date of HBV infection, an individual’s first BC health care record date was used as the start of follow-up since most individuals diagnosed with chronic HBV in Canada are immigrants from HBV-endemic Asia-Pacific countries (18, 19).

### Statistical analysis

Distributions of the following variables were reported for the HCV infected and control groups: age, sex, ethnicity, major mental illness, problematic alcohol use, injection drug use (IDU), cirrhosis, and material and social deprivation (20). Comorbidities were assessed using the Elixhauser comorbidity index (21). Ethnicity was classified using Onomap (22, 23) into the following categories: South Asian (Pakistani, Indian, Bangladeshi, Nepalese, and Sri Lankan), East and South East Asian (Chinese, Japanese, Korean, and South-East Asian), and other (other BC residents). All variables were assessed at baseline (**Supplemental Table 3**).

For each cancer type, the indirect standardization method was used to calculate age and sex standardized incidence ratios (SIR), comparing observed number of cancer cases after HCV infection (or first negative test date for the control group) to the expected number of cancer cases. Expected numbers were derived from cancer counts from the general population of BC between 1990 and 2016 by five-year age groups, sex, and calendar year. The 95% confidence intervals (CIs) for SIRs were calculated by assuming a Poisson distribution for the observed number of cases. SIRs were reported only among individuals age 30 years and older due the low number of events before this age. Results were considered statistically significant if the 95% CI excluded a value of 1.00. SAS software (version 9.4; SAS Institute, Cary, N.C.) was used to perform all statistical analyses.

### Statement of ethics

The study was approved by the Behavioral Research Ethics Board at the University of British Columbia (study id: H15-01776).

## Results

**Figure 1:**
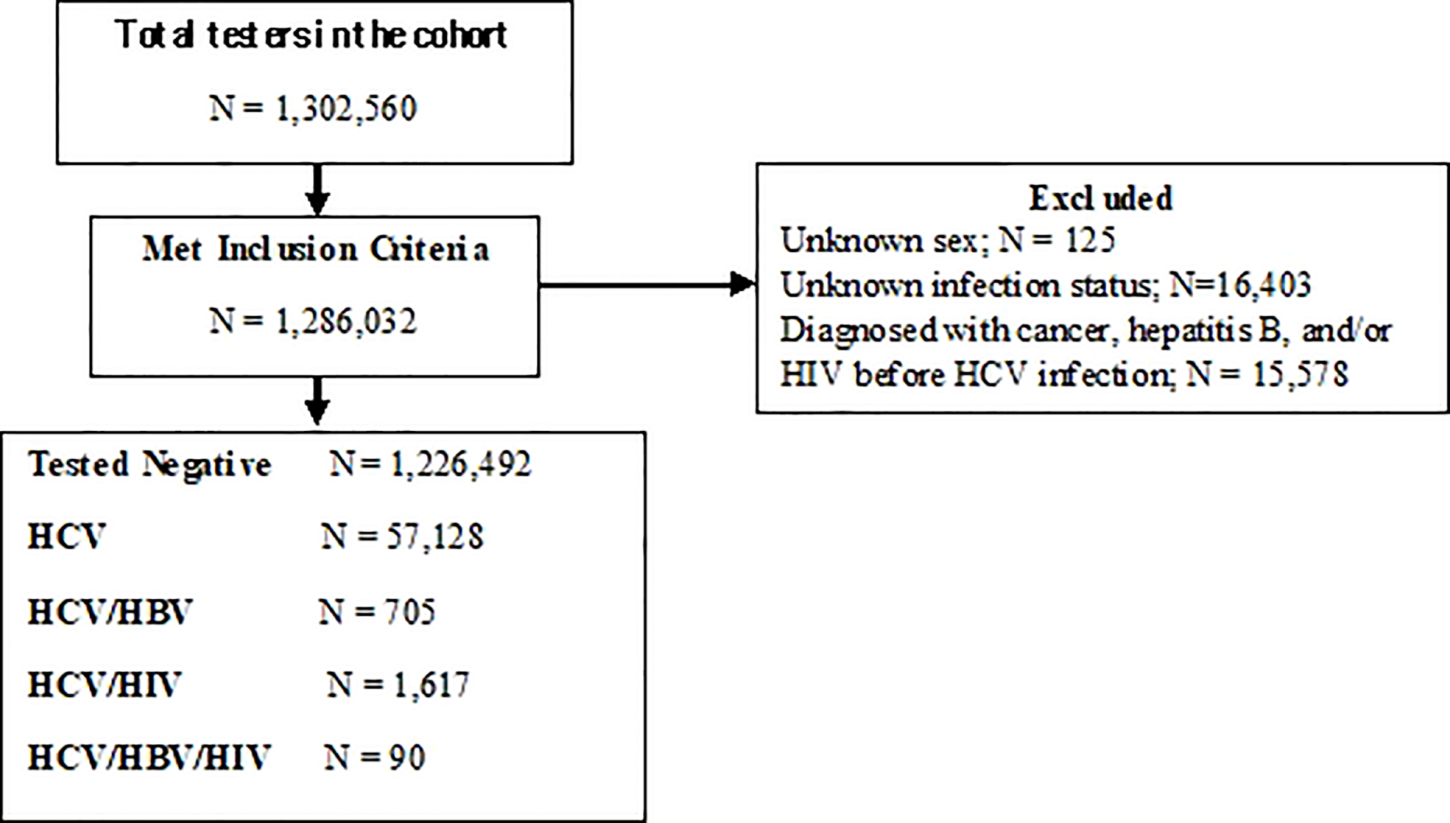
Study Flow diagram.

In total, 1,270,454 individuals were included in the cohort. Among them, 1,207,357 and 56,823 individuals tested negative and positive for HCV infection, respectively (**Table 1**, **Figure 1**). Additionally, 4,453, 1,353, and 468 HCV-infected individuals tested positive for HBV, HIV and HBV/HIV, respectively, after HCV diagnosis (**Table 1**, **Figure 1**). Median age at cancer diagnosis was 59 (interquartile range (IQR): 53-65) among people living with HCV infection, and 63 (IQR: 54-74) in the negative controls. The lowest median age at cancer diagnosis was observed among individuals with HCV/HBV/HIV triple infection (48: IQR: 40-57). A greater proportion of individuals living with HCV were men as compared to negative individuals (66.7% vs. 44.7%, P-value <0.0001). The majority of individuals across infection groups were among the “other” ethnicity category. Compared to the negative group, a higher proportion of individuals living with HCV had comorbidities (25.0% vs. 16.3%, P-value <0.0001) and had a history of injection drug use (1.2% vs. 0.1%, P-value <0.0001) (**Table 1**). For both material and social deprivation, the proportions in the most deprived quintiles were greater for individuals living with HCV infection (socially deprived (35.9% vs. 25.0%, P-value <0.0001), materially deprived (28.6% vs. 18.8%, P-value <0.0001)).

**Table 1:** Baseline characteristics of study participants in BC Hepatitis Testers Cohort, 1990-2015.

Variables	Control N (%)	HCV N (%)	HCV/HBV N (%)	HCV/HIV N (%)	HCV/HBV/HIV N (%)
**^1^Total at baseline 1**	1226492	57128	705	1617	90
**^2^Total at baseline 2**	1207357	56823	4453	1353	468
**Median age at first HCV test/diagnosis date (IQR), years**	37 (27-51)	44 (36-51)	42 (34-50)	39 (33-45)	40 (36-47)
**Median age at cancer diagnosis date (IQR), years**	63 (54-74)	59 (53-65)	57 (51-63)	49 (42-58)	48 (40-57)
**Gender**					
**Male**	548867 (44.7)	38100 (66.7)	473 (67.1)	397 (24.5)	58 (64.4)
**Female**	677625 (55.3)	19028 (33.3)	232 (32.9)	1220 (75.5)	32 (35.6)
**Ethnicity**					
**East Asian**	136979 (11.2)	2208 (3.9)	92 (13.0)	25 (1.5)	2 (2.2)
**Other**	992307 (80.9)	52754 (92.3)	601 (85.3)	1576 (97.5)	88 (97.8)
**South Asian**	97206 (7.9)	2166 (3.8)	12 (1.7)	16 (1.0)	0 (0.0)
Problematic alcohol use*					
**No**	1225218 (99.9)	56895 (99.6)	698 (99.0)	1611 (99.6)	90 (100.0)
**Yes**	1274 (0.1)	233 (0.4)	7 (1.0)	6 (4.0)	0 (0.0)
Major mental illness*					
**No**	1225537 (99.9)	57039 (99.8)	701 (99.4)	1613 (99.7)	89 (98.9)
**Yes**	955 (0.1)	89 (0.2)	4 (0.6)	4 (0.3)	1 (1.1)
Cirrhosis*					
**No**	1225791 (99.9)	57005 (99.8)	703 (99.7)	1617 (0.0)	89 (98.9)
**Yes**	701 (0.1)	123 (0.2)	2 (0.3)	0 (0.0)	1 (1.1)
Elixhauser comorbidity index*					
**No**	1026357 (83.7)	42832 (75.0)	386 (54.7)	809 (50.0)	16 (17.8)
**Yes**	200135 (16.3)	14296 (25.0)	319 (45.3)	808 (50.0)	74 (82.2)
Injection drug use*					
**No**	1225002 (99.9)	56687 (99.2)	695 (98.6)	1598 (98.8)	89 (98.9)
**Yes**	1490 (0.1)	441 (0.8)	10 (1.4)	19 (1.2)	1 (1.1)
Social deprivation*					
**Unknown**	15185 (1.2)	1866 (3.3)	21 (3.0)	44 (2.7)	2 (2.2)
**Q1 (most privileged)**	221030 (18.0)	6182 (10.8)	74 (10.5)	80 (5.0)	4 (4.5)
**Q2**	215947 (17.6)	7298 (12.8)	87 (12.3)	132 (8.1)	4 (4.4)
**Q3**	219346 (17.9)	9409 (16.4)	113 (16.0)	203 (13.0)	8 (8.9)
**Q4**	248593 (20.3)	11868 (20.8)	124 (17.6)	278 (17.2)	15 (16.7)
**Q5 (most deprived)**	306391 (25.0)	20505 (35.9)	286 (40.6)	880 (54.4)	57 (63.3)
Material deprivation*					
**Unknown**	15185 (1.2)	1866 (3.3)	21 (3.0)	44 (2.7)	2 (2.2)
**Q1 (most privileged)**	261658 (21.3)	7249 (12.7)	90 (12.8)	231 (14.3)	15 (16.7)
**Q2**	232283 (18.9)	8888 (15.5)	111 (15.7)	222 (13.7)	11 (12.2)
**Q3**	239652 (19.5)	10093 (17.7)	114 (16.2)	212 (13.1)	11 (12.2)
**Q4**	246833 (20.1)	12704 (22.2)	150 (21.3)	308 (19.1)	20 (22.2)
**Q5 (most deprived)**	230881 (18.8)	16328 (28.6)	219 (31.0)	600 (37.1)	31 (34.5)

^*^At HCV/HBV test or diagnosis date; ^1^Baseline 1: Total at HCV/HBV test or diagnosis date; ^2^Baseline 2: Total at age of 33 for HCV and first BC health care record for HBV.

SIRs were significantly elevated for anal (SIR: 2.57; 95% CI 1.52-3.63), esophageal (SIR: 2.00; 95% CI 1.17-2.82), laryngeal (SIR: 3.24; 95% CI 1.21-5.27), oral (SIR: 1.78; 95% CI 1.33-2.23), lung (SIR: 2.20; 95% CI 1.82-2.58), and liver (SIR 33.09; 95% CI 29.80-36.39) cancers among those with HCV infection (**Table 2**). In sex stratified analyses, the SIR for kidney cancer was elevated among males living with HCV (SIR: 1.74; 95% CI 1.25-2.23), while the SIR for bile duct cancer waselevated among females living with HCV (SIR: 3.05; 95% CI 1.32-4.77) (**Table 2**). The SIRs for cancers of the bile duct and pancreas were significantly elevated among both individuals living with HCV (SIR; 95% CI: 2.20; 1.27-3.14; 2.18; 1.57-2.79, respectively) and the negative control group (SIR; 95% CI: 2.12; 1.88-2.36; 1.20; 1.11-1.28, respectively) (**Table 2**).

**Table 2:** Overall and gender-stratified standardized incidence rates (SIRs) of cancer among negative and HCV mono-infected individuals.

Infection status	Negative SIR (95% CI)				HCV mono-infection SIR (95% CI)			
Cancer Site	Observed* Female/Male	Female	Male	Overall	N Cases Female/Male	Female	Male	Overall
**Anal**	220/139	1.09 (0.90,1.28)	0.99 (0.80,1.19)	1.04 (0.91,1.18)	20/11	3.28 (1.59,4.98)	1.82 (0.59,3.06)	2.57 (1.52,3.63)
**Bile Duct**	426/568	1.99 (1.68,2.29)	2.26 (1.87,2.64)	2.12 (1.88,2.36)	15/21	3.05 (1.32,4.77)	1.31 (0.70,1.92)	2.20 (1.27,3.14)
**Bladder**	965/3154	1.04 (0.94,1.15)	1.14 (1.06,1.22)	1.09 (1.02,1.16)	21/130	1.41 (0.29,2.53)	1.11 (0.77,1.45)	1.26 (0.67,1.86)
**Body of Uterus**	2704	1.10 (1.04,1.15)	NE	NE	20	0.26 (0.12,0.4)	NE	NE
**Breast**	14362/93	1.02 (1.00,1.04)	1.59 (0.45,2.72)	1.30 (0.74,1.85)	236/6	0.63 (0.54,0.73)	1.19 (0.19,2.19)	0.90 (0.41,1.39)
**Cervix**	955	1.03 (0.96,1.10)	NE	NE	22	1.07 (0.43,1.70)	NE	NE
**Colorectal**	4291/5564	0.98 (0.93,1.02)	1.05 (1.00,1.10)	1.01 (0.98,1.05)	100/238	1.12 (0.74,1.49)	1.08 (0.83,1.33)	1.10 (0.87,1.32)
**Oesophagus**	202/502	0.97 (0.67,1.28)	0.76 (0.62,0.89)	0.87 (0.70,1.04)	10/62	1.78 (0.37,3.20)	2.22 (1.40,3.04)	2.00 (1.17,2.82)
**Gallbladder**	165/98	1.14 (0.86,1.42)	1.15 (0.73,1.57)	1.15 (0.90,1.40)	3/8	0.59 (0.00,1.27)	1.85 (0.52,3.17)	1.20 (0.47,1.93)
**Kidney**	920/1593	1.37 (1.26,1.48)	1.19 (1.11,1.27)	1.28 (1.21,1.35)	15/106	0.64 (0.31,0.98)	1.74 (1.25,2.23)	1.17 (0.88,1.47)
**Larynx**	105/495	1.31 (0.89,1.73)	1.06 (0.89,1.23)	1.19 (0.95,1.42)	11/51	4.10 (0.48,7.73)	2.32 (0.67,3.97)	3.24 (1.21,5.27)
**Liver**	334/918	1.07 (0.87,1.28)	0.86 (0.78,0.93)	0.97 (0.86,1.08)	278/1062	42.79 (36.65,48.93)	22.82 (20.88,24.76)	33.09 (29.8,36.39)
**Lung**	3745/3980	0.68 (0.64,0.72)	0.64 (0.59,0.70)	0.66 (0.63,0.69)	252/454	2.47 (1.82,3.13)	1.91 (1.55,2.27)	2.20 (1.82,2.58)
**Melanoma**	1622/1712	0.98 (0.93,1.03)	0.96 (0.91,1.01)	0.97 (0.94,1.00)	29/43	0.79 (0.47,1.11)	0.46 (0.31,0.62)	0.63 (0.45,0.81)
**Oral**	730/1454	1.05 (0.97,1.14)	0.97 (0.90,1.03)	1.01 (0.96,1.06)	35/151	1.97 (1.16,2.79)	1.58 (1.26,1.89)	1.78 (1.33,2.23)
**Ovary**	1248	0.92 (0.86,0.97)	NE	NE	33	0.76 (0.48,1.05)	NE	NE
**Pancreas**	1170/1359	1.07 (0.97,1.18)	1.32 (1.18,1.47)	1.20 (1.11,1.28)	45/87	2.60 (1.52,3.69)	1.73 (1.24,2.22)	2.18 (1.57,2.79)
**Prostate**	12550	NE	1.10 (0.92,1.27)	NE	321	NE	0.50 (0.42,0.57)	NE
**Stomach**	412/828	0.87 (0.76,0.97)	0.85 (0.75,0.95)	0.86 (0.78,0.93)	12/44	0.96 (0.33,1.59)	1.21 (0.56,1.85)	1.08 (0.63,1.53)
**Testis**	531	NE	1.25 (1.08,1.42)	NE	15		1.17 (0.03,2.31)	NE
**Thyroid**	1100/340	1.15 (1.08,1.23)	1.06 (0.95,1.18)	1.11 (1.04,1.18)	14/17	0.67 (0.17,1.18)	1.17 (0.48,1.85)	0.91 (0.49,1.34)
**All other cancers**	3050/3201	0.95 (0.91,0.99)	0.91 (0.86,0.95)	0.93 (0.9,0.96)	102/226	1.36 (1.02,1.7)	1.40 (1.18,1.62)	1.38 (1.17,1.58)

NE: Not estimated as it was either gender-related cancer or had zero counts. *Observed number of cancer cases derived from cancer counts in BC-HTC cohort and expected number of cases from the BC general population between 1990 and 2016.

Among the negative control group, the SIRs for kidney cancer (SIR: 1.28; 1.21-1.35), bladder cancer (SIR: 1.09; 95% CI 1.02-1.16), uterine cancer (SIR: 1.10; 95% CI 1.04-1.15), and thyroid cancer (SIR: 1.11; 95% CI 1.04-1.18) were significantly elevated (**Table 2**). In this group, SIRs for lung (SIR: 0.66; 95% CI 0.63-0.69) and stomach (SIR: 0.86; 95% CI 0.78-0.93) cancers were significantly decreased. In sex stratified analyses, SIRs for colorectal cancer (SIR: 1.05; 95% CI 1.00-1.10) and pancreatic cancer (SIR: 1.20; 95% CI 1.11-1.28) were significantly elevated among males (**Table 2**).

Among individuals living with co-infections and triple-infections, SIRs were reported only for cancer sites with at least 5 cases (**Table 3**). Among males living with HCV/HBV co-infections, significantly increased SIRs were observed for liver cancer (SIR: 64.44; 95% CI 49.06-79.83), lung cancer (SIR: 3.20; 95% CI 1.09-5.30), and oral cancer (SIR: 2.58; 95% CI 1.36-3.80) (**Table 3**). A decreased SIR was observed for breast cancer (SIR: 0.50; 95% CI 0.28-0.72) among females living with HCV/HBV co-infections. SIRs for lung cancer (SIR: 4.08; 95% CI 2.38-5.78) and liver cancer (SIR: 11.37; 95% CI 3.26-19.49) were significantly elevated among males living with HCV/HIV-co-infections (**Table 3**). The SIR for lung cancer was significantly elevated among males living with triple-infections (SIR: 3.67; 95% CI 1.33-6.00).

**Table 3:** Overall and gender-stratified standardized incidence rates (SIRs) of cancer among HCV/HBV and HCV/HIV co-infected and HCV/HBV/HIV triple infected individuals.

Infection status	HCV/HBV SIR (95% CI)				HCV/HIV SIR (95% CI)				HCV/HBV/HIV SIR (95% CI)			
Cancer Site	Observed* Female/Male	Female	Male	Overall	N Cases Female/Male	Female	Male	Overall	N Cases Female/Male	Female	Male	Overall
**Anal**	2/0	3.02 (0.00,7.20)	NE	1.55 (0.00,3.70)	1/4	4.77 (0.00,14.11)	9.7 (0.00,19.47)	7.17 (0.41,13.92)	0/4	NE	33.83 (0.00,68.06)	7.17 (0.41,13.92)
**Bile Duct**	0/4	NE	2.72 (0.05,5.39)	1.32 (0.03,2.62)	0/1	NE	1.71 (0.00,5.07)	0.83 (0.00,2.46)	0/0	NE	NE	NE
**Bladder**	1/7	0.48 (0.00,1.41)	0.61 (0.18,1.04)	0.54 (0.02,1.06)	0/2	NE	0.48 (0.00,1.18)	0.23 (0.00,0.57)	1/1	4.64 (0.00,13.73)	0.55 (0.00,1.63)	2.65 (0.00,7.36)
**Body of Uterus**	2	0.29 (0.00,0.72)	NE	NE	1	1.00 (0.00,2.96)	NE	NE	0	NE	NE	NE
**Breast**	27/0	0.97 (0.54,1.40)	NE	0.50 (0.28,0.72)	3/0	0.21 (0.00,0.46)	NE	0.11 (0.00,0.23)	2/0	0.30 (0.00,0.70)	NE	0.15 (0.00,0.36)
**Cervix**	2	0.85 (0.00,2.03)	NE	0.76 (0.00,1.82)	4	4.29 (0.00,9.42)	NE	NE	4	6.34 (0.00,13.18)	NE	NE
**Colorectal**	6/20	1.02 (0.04,2.00)	0.89 (0.38,1.40)	0.96 (0.40,1.52)	0/10	NE	1.04 (0.37,1.71)	0.50 (0.18,0.83)	0/4	NE	1.96 (0.00,4.04)	0.95 (0.00,1.96)
**Oesophagus**	0/3	NE	0.70 (0.00,1.52)	0.34 (0.00,0.74)	0/3	NE	2.88 (0.00,6.24)	1.40 (0.00,3.03)	0/0	NE	NE	NE
**Gallbladder**	1/2	7.64 (0.00,22.62)	5.56 (0.00,13.36)	6.63 (0.00,15.22)	0/0	NE	NE	NE	0/0	NE	NE	NE
**Kidney**	0/12	NE	2.78 (0.10,5.45)	1.35 (0.05,2.65)	0/1	NE	0.71 (0.00,2.09)	0.34 (0.00,1.02)	0/3	NE	2.31 (0.00,4.93)	1.12 (0.00,2.40)
**Larynx**	2/3	6.11 (0.00,14.59)	8.73 (0.00,24.88)	7.38 (0.00,16.36)	0/3	NE	2.06 (0.00,4.4)	1.00 (0.00,2.14)	0/0	NE	NE	NE
**Liver**	37/138	78.59 (51.93,105.25)	49.47 (35.09,63.85)	64.44 (49.06,79.83)	1/17	8.01 (0.00,23.71)	11.37 (3.26,19.49)	9.64 (0.66,18.63)	0/18	NE	79.44 (0.00,178.39)	38.59 (0.00,86.65)
**Lung**	19/58	3.14 (0.00,6.38)	3.26 (0.61,5.91)	3.20 (1.09,5.3)	9/33	4.04 (1.2,6.88)	4.11 (2.32,5.91)	4.08 (2.38,5.78)	3/11	2.07 (0.00,4.41)	5.36 (1.24,9.47)	3.67 (1.33,6.00)
**Melanoma**	1/2	0.20 (0.00,0.58)	0.17 (0.00,0.40)	0.18 (0.00,0.41)	0/1	NE	0.54 (0.00,1.60)	0.26 (0.00,0.78)	0/1	NE	0.55 (0.00,1.64)	0.27 (0.00,0.8)
**Oral**	1/22	0.55 (0.00,1.63)	2.58 (1.36,3.80)	1.54 (0.73,2.35)	1/6	4.59 (0.00,13.59)	1.02 (0.20,1.83)	2.86 (0.00,7.50)	1/5	4.52 (0.00,13.38)	2.54 (0.30,4.78)	3.56 (0.00,8.24)
**Ovary**	2	0.62 (0.00,1.52)	NE	NE	0	NE	NE	NE	0	NE	NE	NE
**Pancreas**	3/9	1.50 (0.00,3.21)	2.36 (0.61,4.11)	1.92 (0.70,3.14)	0/2	NE	2.15 (0.00,5.70)	1.04 (0.00,2.77)	0/1	NE	1.48 (0.00,4.37)	0.72 (0.00,2.12)
**Prostate**	22	NE	0.42 (0.18,0.65)	NE	6	NE	0.86 (0.00,1.76)	NE	1	NE	0.09 (0.00,0.27)	NE
**Stomach**	2/6	2.50 (0.00,6.05)	1.15 (0.21,2.10)	1.85 (0.00,3.73)	0/1	NE	3.86(0,11.43)	1.88 (0.00,5.55)	0/0	NE	NE	NE
**Testis**	3	NE	1.01 (0.00,2.16)	NE	2	NE	1.02 (0.00,2.44)	NE	1	NE	0.93 (0.00,2.75)	NE
**Thyroid**	4/1	1.65 (0.00,3.40)	0.38 (0.00,1.12)	1.03 (0.06,2.00)	0/1	NE	1.07 (0.00,3.17)	0.52 (0.00,1.54)	1/0	1.39 (0.00,4.11)	NE	0.71 (0.00,2.11)
**All other cancers**	9/21	1.49 (0.49,2.48)	1.81 (0.87,2.75)	1.64 (0.96,2.33)	3/24	1.37 (0.00,2.91)	5.67 (3.14,8.21)	3.46 (1.99,4.93)	4/7	6.89 (0.03,13.76)	3.05 (0.73,5.37)	5.03 (1.32,8.73)

NE: Not estimated as it was either gender-related cancer or had zero counts. *Observed number of cancer cases derived from cancer counts in BC-HTC cohort and expected number of cases from the BC general population between 1990 and 2016.

## Discussion

In this large population-based cohort study, we observed significantly elevated incidence of several extrahepatic cancers among people living with chronic HCV infection, irrespective of coinfection with HBV and/or HIV. Furthermore, the risk of several cancers was increased among those testing negative for infection, potentially due to the presence of cancer-related acquisition risks and/or screening bias among uninfected individuals undergoing repeated testing as compared to the general population.

Our finding on the significant association between chronic HCV and liver cancer (SIR = 33.09) is consistent with the well documented causal relationship between HCV and liver cancer (23–25). As with previous studies, including two large population-based case-control and cohort studies conducted in the USA and Denmark, we observed elevated incidence of pancreatic cancer, kidney cancer, lung cancer, and oropharyngeal cancer among people living with HCV infection (9, 25–30). Among extrahepatic cancers, NHL has been reported to have the strongest association with chronic HCV infection (28, 31, 32). Higher proportions of problematic alcohol consumption, injection drug use, and comorbid medical conditions among individuals living with chronic HCV could partly explain the greater incidence of alcohol and/or tobacco-related cancers, namely oral, larynx, esophagus, lung, kidney, and pancreatic cancers in our cohort (**Table 1**) (27). Although data on smoking status were not available in our study, in general, an important proportion of individuals who use opioids also smoke tobacco, increasing the risk of cancer development (7, 33).

Compared to the general population, elevated risks of several cancer sites were also observed among the negative control group. Shared acquisition risks (e.g., higher alcohol consumption, presence of comorbid conditions among individuals living with HCV infection and negative controls who were repeatedly tested for HCV) may potentially contribute to the observed increased risks of extrahepatic cancers among people testing negative for HCV.

Among negative controls, the risk of solid cancer, including cancer of the bile duct, pancreatic cancer, bladder cancer, uterine cancer, thyroid cancer, and colorectal cancer were elevated (**Table 2**). Previous reports from several epidemiological studies and meta-analyses have found higher incidence for these cancers among individuals living with HCV infection (21, 26–30). Although controls in our study were negative for HCV, HBV, and HIV infections, higher presence of acquisition risks which consequently results in repeated HCV testing and potentially screening bias, could partly contribute to the elevated cancer incidence in this population (13).

Among individuals living with co-infection and triple infection, the number of cases for most cancer sites were small. A lower cancer incidence among this population is expected since the risk of premature death due to multi-comorbidity and disease syndemics is higher among these individuals (7, 34),.

Biological mechanisms underlying elevated risks of extrahepatic cancers among individuals living with HCV infection are not well-understood; for NHL, the process seems to involve direct and indirect oncogenic expressions of HCV proteins (e.g., E2) which causes lymphoproliferative disorders such as B-cell continuous replication (32, 35–37). Chronic kidney disease and oral lichen planus are known HCV-related extrahepatic manifestations which may correspondingly increase the risks of renal and oral cancers among people with HCV infection (35–37). Several studies have provided evidence of HCV RNA replication in extrahepatic tissues (38, 39). In general, expression of HCV proteins (e.g., core protein and/or non-structural protein 3 (NS3)) in non-hepatic tissues, activation of oncogenes through inhibition of DNA repair and tumor-suppressor genes, in addition to chronic inflammation, have been suggested as potential mechanisms through which HCV modulates extrahepatic cancer risk (38, 40).

Using a unique population-based resource, we were able to assess the risk of multiple extrahepatic cancers among people living with HCV infection along with negative controls to assess the potential contributions of screening bias and infection acquisition risks. Our extensive data also allowed us to evaluate risks among those with concurrent HBV and/or HIV infections; however, numbers of cases of many cancers were too low in these groups to generate meaningful results. Furthermore, we used the median age of seroconversion from an analysis of a subset of the cohort to assign the follow-up start date among individuals living with HCV infection, which assumes a more sensible and realistic follow-up time to capture the contribution of HCV to cancer incidence.

In this study we aimed to estimate age and sex standardized incidence ratio for different cancer types. As such, the effects of confounding factors, such as problematic alcohol consumption and socioeconomic status, were not taken into account. However, the potential impact of these factors have been previously reported in studies assessing the risk of cancer among individuals living with HCV (6, 7, 30). Findings from previously conducted BC-HTC studies showed significant associations between HCV infection and risk of pancreatic, liver and colorectal cancers even after adjusting for problematic alcohol use, injection drug use (IDU), and comorbid medical conditions (7). Higher material and social deprivations were observed among individuals living with HCV compared to negative control groups and untreated vs. treated individuals in those studies (6, 7). Similarly, in a study conducted by Nyberg et al., cancer rates remained significantly elevated even after stratification for problematic alcohol consumption, tobacco use, diabetes and BMI (30).

It has been shown that the increase risk of NHL could be reversed among individuals who were treated for chronic HCV infection (41–43). However, DAA therapy only became available in 2014, so its impact on cancer risk will need to be explored in future studies. Misclassification of various ethnic groups is probable as Onomap is not able to identify people of mixed ethnicities, people whose surnames are not specific to ethnic groups, people who adopt surnames of another ethnic group, and people of indigenous ethnicity; because of forced assimilation in Canada during the 18th– 20th centuries, indigenous peoples’ names were routinely changed to biblical or other European names (23, 44).

We observed elevated incidence of several extrahepatic cancers among people living with HCV. Elevated cancer incidence among negative HCV testers does highlight the probable contribution of screening bias and impact of factors associated with HCV infection (e.g., social deprivation) on elevated cancer risks. Early HCV diagnosis and treatment, along with education and public health prevention strategies, are needed to reduce the risk of cancer among people living with HCV infection, and potentially populations who are at higher risk of infection.

## Data availability statement

The data analyzed in this study is subject to the following licenses/restrictions: The data that support the findings of this study are available on request from the corresponding author. The data are not publicly available due to privacy or ethical restrictions.Requests to access these datasets should be directed to mdarvishian@bccrc.ca.

## Ethics statement

The study was approved by the Behavioral Research Ethics Board at the University of British Columbia (study id: H15-01776). Written informed consent for participation was not required for this study in accordance with the national legislation and the institutional requirements.

## Author contributions

Conceived and designed the study: MD, RW, NJ, and PB. Analyzed the data: MD, TT. This article was written by MD, taking into account the comments and suggestions of the co-authors. All co-authors had the opportunity to comment on the analysis and interpretation of the findings and approved the final version for publication. All authors contributed to the article and approved the submitted version.

## Funding

This work was supported by BC Centre for Disease Control and Agencies contributing data to the study and the Canadian Institutes of Health Research [Grant # NHC-348216 and PHE-337680].

## Acknowledgments

We acknowledge the assistance of BCCDC, PHSA Performance measurement and reporting, Information Analysts, Ministry of Health Data Access, Research and Stewardship, & medical services plan (MSP), discharge abstract database (DAD) and Medical Beneficiary and Pharmaceutical Services programme areas, BC Ministry of Health, and BC Cancer Agency and their staff involved in data access and procurement, and data management.

## Conflicts of interest

Author MeK received grants from Roche Molecular Systems, Boehringer Ingelheim, Merck, Siemens Healthcare Diagnostics.

The remaining authors declare that the research was conducted in the absence of any commercial or financial relationships that could be construed as a potential conflict of interest.

## Publisher's note

All claims expressed in this article are solely those of the authors and do not necessarily represent those of their affiliated organizations, or those of the publisher, the editors and the reviewers. Any product that may be evaluated in this article, or claim that may be made by its manufacturer, is not guaranteed or endorsed by the publisher.

## Author disclaimer

All inferences, opinions, and conclusions drawn in this publication are those of the author(s), and do not necessarily reflect the opinions or policies of the British Columbia Ministry of Health.
