# Supplementary material for: Chronic hepatitis C infection is associated with higher incidence of extrahepatic cancers in a Canadian population based cohort
Source: Front Oncol. 2022 Oct 13;12:983238. doi: 10.3389/fonc.2022.983238 (PMC9609415; doi:10.3389/fonc.2022.983238)
Supplement: Supplementary file 1 [file Table_1.docx]

**Table 1.** Criteria and data sources for the BC Hepatitis Testers Cohort (BC-HTC)

| **Criteria for Inclusion in BC-HTC** | |
| --- | --- |
|  | |
| All individuals:   - tested at the BCCDC Public Health Laboratory (BC-PHL ) for HCV or HIV OR - reported to BC public health as a confirmed case of HCV, HIV or AIDS, HBV, or active TB OR - included in BC Enhanced Strain Surveillance System (EHSSS) as an acute HBV or HCV case | |
| All individuals meeting at least one the above criteria were linked internally across all their tests and case reports. Those with a valid personal health number (PHN) were then sent for deterministic linkage with the province-wide Cancer and Ministry of Health (MoH) datasets. | |
| **Provincial Communicable Disease Data Sources:** | **Data Date Ranges:** |
| BC-PHL HIV laboratory testing datasets (tests: ELISA, Western blot, NAAT, p24, culture) | 1988–2015 |
| BC-PHL HCV laboratory tests datasets (tests: antibody, HCV RNA, genotyping) | 1992–2019 |
| HIV/AIDS Information System (HAISYS) (public health HIV/AIDS case reports) | 1980–2015 |
| Integrated Public Health information System (iPHIS) (public health case reports of HCV, HBV, and TB) | 1990–2015 |
| Enhanced Strain Surveillance System (EHSSS) (risk factor data on a subset of acute HCV and acute HBV cases) | 2000–2013 |
| **Cancer and MoH Administrative Data Sources:** | **Data Date Ranges:** |
| BC Cancer Registry (BCCR) (primary tumour registry, excludes metastatic cancers) | 1923–2016 |
| Client Roster (CR) (Registry of enrollment in the universal public health insurance plan including residential history) ^S1^ | 1990–2016 |
| Discharge Abstracts Dataset (DAD) (hospitalization records) ^S2^ | 1985–2015 |
| Medical Services Plan (MSP) (physician diagnostic and billing data) ^S3^ | 1990–2015 |
| PharmaCare/PharmaNet (Pharma) (prescription drug dispensations) ^S4, S5^ | 1985–2019 |
| BC Vital Statistics (VS) (deaths registry)^S6^ | 1985–2019 |
| NACRS (Emergency Departments) ^S7^ | 2012–2015 |
| Chronic Disease Registry^S8^ | 1992–2015 |
| The final BC-HTC comprises all individuals successfully linked on PHN to the MoH Client Roster^S1^ (a registry of all BC residents enrolled in the publicly-funded universal healthcare system) | |

HCV: Hepatitis C Virus; HBV: Hepatitis B Virus; HIV/AIDS: Human Immunodeficiency Virus/Acquired Immunodeficiency Syndrome; BC-PHL: BC Public Health Laboratory: RNA: Ribonucleic Acid; PCR: Polymerase Chain Reaction.

**Supplementary References**:

1. British Columbia Ministry of Health [creator]. Discharge Abstract Database (Hospital Separations). British Columbia Ministry of Health [publisher]. Data Extract. MOH (2013). 2016. <http://www.health.gov.bc.ca/data/>
2. British Columbia Ministry of Health [creator]. Medical Services Plan (MSP) Payment Information File. British Columbia Ministry of Health [publisher]. Data Extract. MOH (2013). 2016. <http://www.health.gov.bc.ca/data/>
3. British Columbia Ministry of Health [creator]. PharmaCare. British Columbia Ministry of Health [publisher]. Data Extract. MOH (2013). July 2017. <http://www.health.gov.bc.ca/data/>
4. British Columbia Ministry of Health [creator]. PharmaNet. British Columbia Ministry of Health [publisher]. Data Extract. MOH (2013). July 2017. <http://www.health.gov.bc.ca/data/>
5. BC Vital Statistics Agency [creator]. Vital Statistics Deaths. BC Vital Statistics Agency [publisher]. Data Extract. BC Vital Statistics Agency (2014). 2017.

British Columbia Ministry of Health [creator]. Client Roster (Client Registry System/Enterprise Master Patient Index). British Columbia Ministry of Health [publisher]. Data Extract. MOH (2013). 2016. <http://www.health.gov.bc.ca/data/>

**Table 2.** Cancer site groupings.

| **Cancer** | **ICD-O-3 Site/Type (incidence)** |
| --- | --- |
| Anal | C21 |
| Bile duct | C24 |
| Bladder | C67 |
| Body of uterus | C54–C55 |
| Breast | C50 |
| Cervix | C53 |
| Colorectal | C18–C20, C26.0 |
| Oesophagus | C15 |
| Gallbladder | C25 |
| Kidney | C64.9, C65.9 |
| Larynx | C32 |
| Liver | C22 |
| Lung | C34 |
| Melanoma | C44 (Type 8720–8790) |
| Oral | C00-C14 |
| Ovary | C56.9 |
| Pancreas | C25 |
| Prostate | C61.9 |
| Stomach | C16 |
| Testis | C62 |
| Thyroid | C73.9 |
| All other cancers | All invasive sites |

**Table 3**. Administrative code and definitions.

| **Diabetes mellitus (DM)**  Diabetes mellitus was defined at the occurrence of the second of 2 MSP, within one year, or 1 hospitalization diagnostic code for diabetes mellitus, or the prescription of at least two oral hypoglycemic drugs or insulin within one year.  **Physician Billing Data**: MSP ICD-9 diagnostic codes: starting with 250.  **Hospitalization Data**: DAD1/ICD-9-CM: starting with 250; DAD2/ICD-10-CA: starting with E10, E11, E12, E13 or E14.  **PharmaNet Data:** DINPIN numbers: 5894, 6009, 12556, 12564, 12599, 12602, 12610, 13730, 13889, 15598, 21350, 21849, 24708, 24716, 93033, 156663, 156728, 178543, 209872, 209937, 237000, 244449, 271330, 274119, 274127, 275409, 275417, 275425, 312711, 312762, 314552, 377937, 399302, 420336, 430986, 431168, 446564, 446572, 446580, 446599, 446602, 446610, 454753, 480290, 480304, 513644, 514535, 514551, 539201, 539244, 542911, 542938, 542946, 546348, 552259, 552267, 552275, 554820, 586714, 586773, 587737, 612162, 612170, 612189, 612197, 612200, 612219, 612227, 612235, 612243, 612251, 612278, 612359, 614416, 628301, 632651, 632678, 632686, 632694, 644358, 646148, 648094, 650935, 720933, 720941, 723789, 733075, 765996, 773654, 795879, 808733, 808741, 889091, 889105, 889113, 889121, 999717, 999814, 1900927, 1900935, 1913654, 1913662, 1913670, 1913689, 1934066, 1934074, 1934082, 1934090, 1934104, 1934112, 1959212, 1959220, 1959239, 1959352, 1959360, 1962639, 1962647, 1962655, 1962663, 1985930, 1985949, 1985957, 1985965, 1985973, 1985981, 1986085, 1986791, 1986805, 1986813, 1986821, 1987534, 1987542, 1987828, 1987836, 2020734, 2020742, 2022230, 2022249, 2024217, 2024225, 2024233, 2024241, 2024268, 2024276, 2024284, 2024292, 2024306, 2024314, 2024322, 2024403, 2024446, 2025248, 2025256, 2045710, 2084341, 2085887, 2099233, 2147521, 2147548, 2148765, 2155850, 2162822, 2162849, 2167786, 2188902, 2190885, 2190893, 2220628, 2223562, 2224550, 2224569, 2224771, 2224798, 2226804, 2226812, 2228920, 2228939, 2229516, 2229517, 2229519, 2229595, 2229596, 2229656, 2229704, 2229705, 2229785, 2229994, 2230026, 2230027, 2230036, 2230037, 2230443, 2230444, 2230475, 2230670, 2230671, 2231058, 2231095, 2231096, 2231389, 2233562, 2233999, 2234513, 2234514, 2236543, 2236548, 2236733, 2236734, 2236985, 2236986, 2237531, 2238103, 2238469, 2238470, 2238471, 2238698, 2238827, 2239081, 2239214, 2239474, 2239475, 2239476, 2239924, 2239925, 2239926, 2240294, 2240295, 2240297, 2241111, 2241112, 2241113, 2241114, 2241283, 2241310, 2242095, 2242096, 2242572, 2242573, 2242574, 2242589, 2242726, 2242783, 2242793, 2242794, 2242931, 2242974, 2242987, 2244353, 2245247, 2245272, 2245273, 2245274, 2245397, 2245438, 2245439, 2245440, 2245689, 2246820, 2246821, 2246964, 2246965, 2247085, 2247086, 2247087, 2248008, 2248009, 2248210, 2248440, 2248441, 2248453, 2251930, 2252945, 2252953, 2254719, 2257726, 2257734, 2258781, 2258803, 2258811, 2265435, 2265443, 2265575, 2265583, 2268493, 2268507, 2269031, 2269058, 2269589, 2269597, 2269600, 2269619, 2271842, 2273101, 2273128, 2273136, 2273756, 2273764, 2273772, 2274248, 2274256, 2274264, 2274272, 2274914, 2274922, 2274930, 2275864, 2275872, 2276410, 2279061, 2279088, 2279126, 2279460, 2279479, 2279487, 2284545, 2284553, 2284782, 2284790, 2287072, 2294338, 2294346, 2294400, 2295377, 2295385, 2295393, 2297795, 2297906, 2297914, 2297922, 2298279, 2298287, 2298295, 2300451, 2301423, 2301431, 2301458, 2302861, 2302888, 2302896, 2302942, 2302950, 2302977, 2303124, 2303132, 2303140, 2303442, 2303450, 2303469, 2303922, 2305062, 2306166, 2306174, 2306182, 2307170, 2307189, 2307197, 2307553, 2307561, 2307588, 2307634, 2307642, 2307650, 2307669, 2307677, 2307723, 2312050, 2312069, 2312077, 2313596, 2314894, 2314908, 2316544, 2320754, 2320762, 2320770, 2321475, 2321483, 2321491, 2326329, 2326337, 2326345, 2326477, 2326485, 2326493, 2331519, 2331527, 2333554, 2333856, 2333864, 2333872, 2334437, 2334445, 2336316, 2339110, 2339129, 2339587, 2339595, 2340763, 2340771, 2341522, 2341603, 2343606, 2343614, 2345366, 2345374, 2345382, 2345854, 2345862, 2348578, 2350459, 2350467, 2351056, 2351064, 2353377, 2353385, 2354144, 2354152, 2354160, 2354349, 2354357, 2354365, 2354926, 2354934, 2354942, 2355663, 2355671, 2355698, 2356422, 2357453, 2357461, 2357488, 2357887, 2357895, 2357909, 2357917, 2357925, 2361264, 2361272, 2361809, 2361817, 2363232, 2363240, 2363259, 2363518, 2363704, 2363712, 2364506, 2364514, 2365286, 2365294, 2365529, 2365537, 2366347, 2366355, 2366363, 2370921, 2373270, 2373289, 2373297, 2374013, 2374021, 2374048, 2374587, 2374595, 2375842, 2375850, 2375869, 2375877, 2377209, 2378043, 2378051, 2378116, 2378124, 2378620, 2378639, 2378841, 2378868, 2379767, 2379775, 2380196, 2380218, 2380722, 2380730, 2384906, 2384914, 2384922, 2385341, 2385368, 2388766, 2388774, 2388839, 2388847, 2389169, 2389177, 2389185, 2389290, 2389304, 2389312, 2391600, 2397307, 2403250, 2403269, 2403277, 2403366, 2403374, 2403382, 2403412, 2403420, 2403439, 2403447, 2405067, 2406020, 2406039, 2408228, 2408236, 2409283, 2409291, 22303140, 45230001, 45230002, 45230003, 45230004, 45230005, 45230006, 45230007, 45230008, 45230009, 45230010, 47450001,47450002, 47450003, 47450004, 47450005, 47450006, 47450007. |
| --- |
| **Injection drug use (IDU)**  Injection drug use was defined at the occurrence of 2 MSP or 1 hospitalization diagnostic code for major drug-related diagnoses involving addiction, dependence, and drug-induced mental disorders; illicit drug use most likely to be injectables (e.g. excluding cannabis), or illicit use of prescribed drugs including: hallucinogens, barbiturates/tranquillizers, sedatives, hypnotics, anxiolytics, opioids, cocaine, amphetamine, volatile solvents; or discharge to drug rehabilitation, counselling, and surveillance.  **Physician Billing Data**: MSP ICD-9 diagnostic codes: starting with 292, 3040, 3042, 3044, 3046-9, 3054-7, 3059, 6483, 9650, 9658, 9663-64, 9670, 9684, 9685, 9697-99, 9700-01, 9708-09, E8500, or exact codes V6542 or fee item = 39  **Hospitalization Data**: DAD1/ICD-9-CM: starting with 292, 3040-42, 3044-49, 3054-7, 3059, 6483, 7960, 9621, 9650, 9658, 9663-64, 9670, 9684-85, 9694, 9696-99, 970, 9700-01, 9709-09, E8500, V6542; DAD2/ICD-10-CA: starting with F11, F13-5, F19, or exact codes R781-82, T387, T400-T406, T409, T412, T423-28, T436-439, T507. |
| **Problematic alcohol consumption**  Problematic alcohol use was defined at the first occurrence of 2 MSP or 1 hospitalization codes for major alcohol-related diagnoses including alcoholic mental disorders and dependence/abuse syndromes; alcoholic polyneuropathy, myopathy, cardiomyopathy; pseudo Cushing’s syndrome; or discharge to alcohol rehabilitation, counselling, or surveillance  **Physician Billing Data:** MSP ICD-9 diagnostic codes: starting with 291, 303, 3050, 3575, 4255  **Hospitalization Data:** DAD1/ICD-9-CM: starting with 291, 303, 3050, 3575, 4255; DAD2/ICD-10-CA: starting with F10, E244, G312, G621, G721, I426, Z502, Z714 |
| **Hepatitis B virus (HBV) co-infection**  HBV coinfection was defined at the first occurrence of 2 MSP or 1 hospitalization for HBV, 2 MSP fee item codes for HBV DNA or HBV e-antigen testing, 1 dispensation for HBV-specific antiviral treatments, or a confirmed public health case report recorded in the integrated Public Health Information System (iPHIS) defined on the basis of provincial guidelines.  **Physician Billing Data:** MSP ICD-9 diagnostic codes: starting with 702, 703, V0261; Fee item codes: 90675, 90690, 90831, 91765.  **Hospitalization Data:** DAD1/ICD-9-CM: starting with 702, 703, V0261; DAD2/ICD-10-CA: starting with B16, B180, B181, Z2250.  **PharmaNet Data:** DINPIN numbers: 02239193, 02247128, 02247823, 02282224, 02288389. |
| **Human immunodeficiency virus (HIV) co-infection**  HIV coinfection was defined at the first occurrence of 3 MSP or 1 hospitalization for HIV, or a positive HIV serologic test, HAISYS or BC Vital Statistics indication.  **Physician Billing Data:** MSP ICD-9 diagnostic codes: starting with 042, 043, 044, 7953, 7958, 79571, V08.  **Hospitalization Data:** DAD1/ICD-9-CM: starting with 042, 043, 044, 7953, 7958, 79571, V08; DAD2/ICD-10-CA: starting with B20-B24, B9735, F024, O987, R75, Z21. |
| **Liver cirrhosis**  Liver cirrhosis was defined as the first occurrence of 2 MSP or 1 hospitalization with the liver disease-related diagnostic codes shown below.  **Physician Billing Data:** MSP ICD-9 diagnostic codes: starting with 070.44, 456, 567.23, 571.2, 571.3, 571.5, 572.2, 572.3, 572.4, 572.8, 789.5.  **Hospitalization Data:** DAD1/ICD-9-CM: starting with 070.44, 456, 567.23, 571.2, 571.3, 571.5, 572.2, 572.3, 572.4, 572.8, 789.5; DAD2/ICD-10-CA: starting with I85, I98.20, I98.3, K65.2, K72.1, K72.9, K76.6, K70.3, K76.7, K7460, K7469, K704, R18. |
| **Elixhauser Comorbidity Score**  Using DAD ICD-9 and ICD-10 code data, we calculated a score in which any hospitalization for one of the 31 Elixhauser diagnostic groups was scored as 1.  Congestive Heart Failure, Cardiac Arrhythmia, Valvular Disease, Pulmonary Circulation Disorders, Peripheral Vascular Disorders, Hypertension Uncomplicated, Hypertension Complicated, Paralysis, Neurological Disorders, Chronic Pulmonary Disease, Diabetes Uncomplicated, Diabetes Complicated, Hypothyroidism, Renal Failure, Liver Disease, Peptic Ulcer Disease excluding bleeding, AIDS/HIV, Lymphoma, Metastatic Cancer, Solid Tumor without Metastasis, Rheumatoid Arthritis/collagen, Coagulopathy, Obesity, Weight Loss, Fluid and Electrolyte Disorders, Blood Loss Anemia, Deficiency Anemia, Alcohol Abuse, Drug Abuse, Psychoses, Depression. |
| **Material and Social Deprivation Quintiles**  The Québec Index of Material and Social Deprivation was calculated based on individuals’ 6-digit postal code. The deprivation index combines six indicators related to health and welfare that represent material or social deprivation and are available by enumeration area in Canadian census data: 1) proportion of persons without high-school diploma 2) ratio of employment to population 3) average income 4) proportion of persons separated, divorced, widowed 5) the proportion of single-parent families 6) proportion of people living alone. |

Abbreviations: DAD: discharge abstracts database; DIN: drug identification number; ICD: international classification of diseases; MSP: medical services plan; PIN: product information number.

**Table 4**. Overall and gender-stratified standardized incidence rates (SIR) of cancer among negative and HCV mono-infected individuals at HCV/HBV test/diagnosis date.

| Infection status | Negative  SIR (95% CI) | | | | HCV mono-infection  SIR (95% CI) | | | |
| --- | --- | --- | --- | --- | --- | --- | --- | --- |
| Cancer Site | **N Cases Female/Male** | **Female** | **Male** | **Overall** | **N Cases Female/Male** | **Female** | **Male** | **Overall** |
| Anal | 123/73 | 1.07 (0.87, 1.28) | 1.11 (0.80, 1.42) | 1.09 (0.91,1.27) | 20/11 | 3.22 (1.56, 4.87) | 1.79 (0.57, 3.00) | 2.52 (1.49, 3.56) |
| Bile Duct | 376/501 | 3.44 (2.86, 4.03) | 4.38 (3.51, 5.26) | 3.90 (3.38, 4.42) | 15/23 | 3.00 (1.30, 4.71) | 1.39 (0.78, 2.00) | 2.22 (1.29, 3.15) |
| Bladder | 494/1461 | 0.95 (0.81, 1.09) | 1.02 (0.90, 1.14) | 0.98 (0.89, 1.08) | 22/134 | 1.41 (0.32, 2.50) | 1.11 (0.78, 1.44) | 1.26 (0.68, 1.85) |
| Body of Uterus | 1215 | 1.00 (0.92, 1.08) | - | - | 21 | 0.27 (0.13, 0.41) | - | - |
| Breast | 6600 | 0.94 (0.92, 0.97) | 1.08 (0.60, 1.56) | 1.01 (0.78, 1.24) | 248 | 0.66 (0.56, 0.76) | 1.17 (0.18, 2.16) | 0.91 (0.43, 1.39) |
| Cervix | 363 | 0.73 (0.65, 0.81) | - | - | 21 | 1.02 (0.39, 1.65) | - | - |
| Colorectal | 2234/2781 | 1.01 (0.95, 1.08) | 1.17 (1.08, 1.25) | 1.09 (1.03, 1.14) | 104/243 | 1.13 (0.76, 1.49) | 1.05 (0.81, 1.29) | 1.09 (0.87, 1.31) |
| Oesophagus | 152/378 | 1.50 (0.88, 2.11) | 1.18 (0.95, 1.42) | 1.34 (1.01, 1.68) | 10/63 | 1.75 (0.36, 3.13) | 2.20 (1.40, 3.00) | 1.97 (1.16, 2.78) |
| Gallbladder | 137/83 | 2.02 (1.44, 2.60) | 1.92 (1.16, 2.68) | 1.97 (1.50, 2.45) | 2/9 | 0.40 (0.00, 0.95) | 2.04 (0.49, 0.67) | 1.19 (0.47, 1.92) |
| Kidney | 482/795 | 1.31 (1.16, 1.46) | 1.30 (1.17, 1.45) | 1.31 (1.21, 1.41) | 15/107 | 0.63 (0.30, 0.96) | 1.70 (1.23, 2.18) | 1.15 (0.86, 1.44) |
| Larynx | 53/225 | 1.47 (0.78, 2.17) | 0.94 (0.69, 1.19) | 1.21 (0.84, 1.59) | 12/51 | 4.21 (0.65, 7.76) | 2.27 (0.67, 3.87) | 3.26 (1.28, 5.25) |
| Liver | 258/692 | 1.64 (1.26, 2.02) | 1.39 (1.23, 1.55) | 1.52 (1.31, 1.73) | 290/1106 | 43.68 (37.59, 49.78) | 23.57 (21.53, 25.61) | 33.91 (2.81, 30.63) |
| Lung | 2826/3002 | 0.93 (0.87, 0.99) | 0.98 (0.88, 1.08) | 0.96 (0.90, 1.01) | 256/457 | 2.59 (1.89, 3.30) | 1.84 (1.50, 2.19) | 2.23 (1.83, 2.63) |
| Melanoma | 821/745 | 1.01 (0.94, 1.08) | 0.86 (0.79, 0.93) | 0.94 (0.89, 0.99) | 29/43 | 0.77 (0.46, 1.09) | 0.46 (0.30, 0.61) | 0.62 (0.44, 0.80) |
| Oral | 420/780 | 1.18 (1.05, 1.31) | 1.08 (0.98, 1.19) | 1.33 (1.05, 1.22) | 35/157 | 1.93 (1.13, 2.72) | 1.59 (1.28, 1.91) | 1.77 (1.33, 2.20) |
| Ovary | 699 | 0.97 (0.89, 1.06) | - | - | 33 | 0.75 (0.47, 1.03) | - | - |
| Pancreas | 1040/1220 | 1.90 (1.69, 2.12) | 2.69 (2.36, 3.02) | 2.29 (2.09, 2.48) | 44/87 | 2.52 (1.46, 3.58) | 1.69 (1.21, 2.17) | 2.12 (1.53, 2.71) |
| Prostate | 5575 | - | 1.15 (0.87, 1.43) | - | 327 | - | 0.49 (0.42, 0.57) | - |
| Stomach | 281/543 | 1.12 (0.94, 1.30) | 1.15 (0.98, 1.33) | 1.14 (1.01, 1.26) | 12/43 | 0.94 (0.32, 1.55) | 1.16 (0.54, 1.79) | 1.05 (0.61, 1.49) |
| Testis | 174 | - | 1.04 (0.80, 1.29) | - | 15 | - | 1.14 (0.03, 2.26) | - |
| Thyroid | 612/184 | 1.34 (1.23, 1.46) | 1.34 (1.14, 1.54) | 1.31 (1.23, 1.46) | 15/18 | 0.71 (0.20, 1.21) | 1.17 (0.49, 1.84) | 0.93 (0.51, 1.35) |
| All other cancers | 1961/1991 | 1.08 (1.02, 1.14) | 1.10 (1.03, 1.17) | 1.09 (1.04, 1.14) | 103/230 | 1.34 (1.01, 1.67) | 1.39 (1.17, 1.60) | 1.36 (1.16, 1.56) |
